# Exercise Training in Treatment and Rehabilitation of Hip Osteoarthritis: A 12-Week Pilot Trial

**DOI:** 10.1155/2017/3905492

**Published:** 2017-01-01

**Authors:** Kirsti Uusi-Rasi, Radhika Patil, Saija Karinkanta, Kari Tokola, Pekka Kannus, Harri Sievänen

**Affiliations:** ^1^The UKK Institute for Health Promotion Research, Tampere, Finland; ^2^Department of Orthopaedics and Trauma Surgery, Tampere University Hospital and Medical School, University of Tampere, Tampere, Finland

## Abstract

*Introduction. *Osteoarthritis (OA) of the hip is one of the major causes of pain and disability in the older population. Although exercise is an effective treatment for knee OA, there is lack of evidence regarding hip OA. The aim of this trial was to test the safety and feasibility of a specifically designed exercise program in relieving hip pain and improving function in hip OA participants and to evaluate various methods to measure changes in their physical functioning.* Materials and Methods*. 13 women aged ≥ 65 years with hip OA were recruited in this 12-week pilot study.* Results*. Pain declined significantly over 30% from baseline, and joint function and health-related quality of life improved slightly. Objective assessment of physical functioning showed statistically significant improvement in the maximal isometric leg extensor strength by 20% and in the hip extension range of motion by 30%.* Conclusions*. The exercise program was found to be safe and feasible. The present evidence indicates that the exercise program is effective in the short term. However, adequate powered RCTs are needed to determine effects of long-term exercise therapy on pain and progression of hip OA.

## 1. Introduction

Osteoarthritis (OA) is a common disease presenting with joint pain, stiffness, swelling, and instability resulting in functional impairment in daily activities. Due to its high prevalence in the older population, OA has a major impact on healthcare costs globally. Pharmacological treatment is not recommended as the primary treatment for OA [1–3], and effects of various physical therapy techniques on relieving pain or improving joint function have remained rather small [4]. Consequently, symptomatic hip OA often leads to hip replacement surgery.

The main treatment goal in OA is to reduce joint pain and minimize physical disability [5]. Effectiveness of aerobic and strength training is recommended as the first-line conservative treatment approach in adults with mild-to-moderate knee OA [3]. Despite current national and international guidelines for the use of exercise in patients with hip OA, very few clinical exercise trials have been conducted in patients with hip OA. A Cochrane review by Fransen et al. included 10 exercise trials [6], and only 5 recruited solely patients with hip OA [7–10]; one of these was presented as an abstract only [11]. Five other included studies had mixed sample of hip and knee OA patients with the proportion of hip OA in these combined programs being always smaller than the proportion with knee OA [12–16]. However, the results are inconsistent.

Programs developed for OA of the lower limbs seem to benefit patients with knee OA more than those with hip OA. Juhl et al. found that exercise programs for knee OA should focus on improving aerobic capacity, quadriceps muscle strength, or lower extremity performance. For optimal results, the program should be supervised and carried out 3 times weekly and comprise at least 12 sessions [17].

Exercise therapy aims at reducing pain and disability by improving muscle strength, joint stability, range of motion (ROM), and aerobic fitness [10]. Whereas training focusing on improved muscle strength and aerobic capacity is known to alleviate OA symptoms, effects of exercise need further elucidation [18]. Patients with hip OA are assumed to respond to exercise in the same way as patients with other chronic lower limb pain conditions do. Hip OA patients need specifically developed and executed exercise training to ensure adequate compliance [19].

Thus, more effective, feasible, and sustainable exercise protocols for hip OA are needed for further developing therapeutic exercise recommendations for the disease. The present 12-week pilot trial aimed to test the safety and feasibility of a specifically designed exercise program in relieving hip pain and improving function in hip OA subjects and to evaluate methods to measure changes in physical functioning.

## 2. Participants and Methods

### 2.1. Participants

Participants were recruited from the waiting list of the orthopedic outpatient clinic of Hatanpää and COXA Hospital (specialized in joint replacements) in Tampere, Finland. Thirteen women aged between 65 and 83 years, with moderate or severe restrictions in mobility, debilitating pain, and difficulties in walking, stair climbing, or putting on shoes, volunteered to participate in this pilot trial and gave informed consent. A health history questionnaire screened for self-reported health, comorbidities, medication, and lifestyle (physical activity, use of alcohol, and smoking). Participants were then invited to a baseline examination, which included a physician's examination, questionnaires, and measurements of physical functioning (strength, balance, and mobility).

Inclusion criteria were age ≥ 65 years, living at home independently, and unilateral or bilateral hip OA with pain in the hip region (groin and lateral hip) during the preceding month. Exclusion criteria were bilateral total hip replacement, moderate-to-severe knee OA, fracture during the preceding 12 months, and chronic conditions such as rheumatoid arthritis or major surgical procedures in the preceding 6 months (lower limb or lower back). Medication used was not an inclusion or exclusion criterion.

This study was conducted according to the guidelines of good clinical practice, and the study protocol was approved by the Pirkanmaa Hospital District Ethics Committee, Tampere, Finland (R15004).

### 2.2. Anthropometry

Height and weight were measured with standard methods. Body composition (fat and lean soft tissue mass) and femoral neck bone mineral density were assessed with dual-energy X-ray absorptiometry (DXA, Lunar Prodigy Advance, GE Lunar, Madison, WI, USA) [20]. DXA measurement was performed only at baseline. All other measurements described below were done at baseline and at 12 weeks.

### 2.3. Pain and Self-Reported Physical Function

The primary outcome of the study was hip joint pain assessed by the Western Ontario and McMaster University Osteoarthritis Index [21] (WOMAC, Finnish version [22]). WOMAC produces three subscale scores (pain, stiffness, and physical function) and a total score (WOMAC Index) that reflects overall disability. Each item is assessed on a Visual Analog Scale, with a possible range of scores of 0–100 mm. Items are summed for each subscale, pain (range = 0–500 mm, 5 items), stiffness (range = 0–200 mm, 2 items), and physical function (range = 0–1700 mm, 17 items), and for the total WOMAC Index (range: 0–2400 mm). Self-reported disease-specific disability was assessed using the pain and functioning subscales at baseline and at 12 weeks [23]. Quality of life was assessed by the LEIPAD questionnaire [24].

### 2.4. Hip Joint Assessment and Physical Functioning

Physical functioning (strength, balance, and mobility) was measured objectively. The maximal isometric leg extensor muscle strength was measured by a leg press dynamometer. Timed-Up and Go (TUG) [25], the Short Physical Performance Battery (SPPB) (static balance, 4-meter walking speed and five-time chair stand) [26], 9-step stair climb 20 cm [27], and hip ROM [28] were assessed. Postural balance was assessed using the force platform (Good Balance, Metitur, Jyväskylä, Finland) [29]. The system uses vertical force signals from each corner of the platform to calculate* x* (mediolateral, ML) and* y* (anteroposterior, AP) coordinates of the platform center of pressure (COP) when the test person stood on it. Mean ML and AP velocity (mm/s) and moment of velocity (mm^2^/s) were calculated. Balance was tested in the normal standing position in four test conditions: eyes open, eyes closed, eyes open with cognitive task (mental arithmetic), and eyes open while standing on a foam sheet. Pedometers (Omron WS III; Omron Healthcare, Inc., Lake Forest, IL) were used throughout the 12-week period for objective assessment of daily steps taken.

### 2.5. Training Program

Training was led or implemented as circuit training sessions by experienced exercise leaders (physiotherapists) 3 times a week for 12 weeks. Five sessions were offered weekly, from which participants could select any three. Training was started with a 2-week familiarizing period to accustom the participants to the exercise, followed by 5 weeks in the exercise hall and 5 weeks in the gym. All sessions lasted 60 minutes and included a 10-minute warm-up as well as stretching for major muscle groups. Exercise leaders kept a record of participants' attendance and possible adverse events.

Training was progressive and was implemented as group-based sessions but was planned with individual goals and limitations in mind. Sessions in the exercise hall focused on range of motion, lower limb muscle strength, balance, agility, mobility, and change of direction. Progression was achieved with the use of different surfaces, multidirectional movement patterns, and changing the base of support. In addition to own body weight, ankle or vest weights and step-boards of increasing height were used to increase the intensity of training. Advanced programs were also aerobic in nature.

During the gym sessions, resistive equipment was used. All sessions included 8-9 different exercises focusing on strengthening lower limb muscles (leg extensors, hip extensors, hip abductors, hip rotators, knee extensors, and calf muscles) as well as other large muscle groups (abdominal, back, shoulder, and arm muscles). The first gym period began with 30–60% of one repetition maximum (1RM) progressing to 60–75% of 1RM over 5 weeks. Two sets of each exercise were done, with each set consisting of 8–12 repetitions. Intensity of training was assessed using the rate of perceived exertion scale (RPE). The target RPE ranged from 13 to 18 and advanced progressively. Balance training was included in a short warm-up period. Detailed description of the training program is presented in Table 2.

### 2.6. Statistical Analysis 

Descriptive information is presented as means and standard deviations (SD). Paired* t*-tests were used to compare changes over time (12 weeks) in pain and physical functioning. Results related to WOMAC scores, physical functioning, and quality of life are presented as percent changes with 95% confidence intervals (CI). *p* values less than 0.05 were considered statistically significant. Because the purpose of this pilot study was to test the feasibility and safety of the exercise program, power calculations for treatment effects were not done.

## 3. Results

Baseline characteristics are given in Table 1. All participants were nonsmoking women with mean age (SD) of 71.6 (6.0) years. Mean height was 163.5 (7.0) cm, weight was 76.5 (12.3) kg, and body mass index (BMI) was 28.5 (3.3) kg/m^2^. Weight remained constant [mean change: 0.1 (1.9) kg, *p* = NS] during the 12-week intervention. Three women had no diagnosed illness other than hip OA, and the most common medication was for high blood pressure (*n* = 8). No changes were made in OA medication during the intervention. The most often used medication was the NSAIDs (nonsteroid anti-inflammatory drugs).

### 3.1. Safety and Feasibility of the Program

Exercise compliance measured as attendance at all offered sessions was 90% (range: 42% to 100%), and all participants attended the end point measurements. In general, the training program was well tolerated and no one consulted the attending physician (PK), although one participant withdrew from the training due to back pain (additional diagnosis of prolapsus disci intervertebralis was done during the intervention).

### 3.2. Effects on Pain, Stiffness, and Function

**Figure 1:**
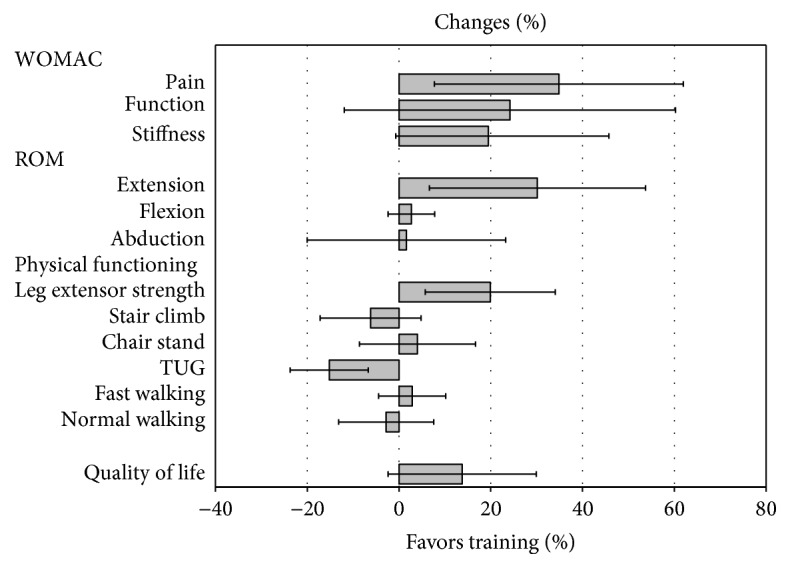
Mean changes (95% CI) in the main outcome variables in 12 weeks.

**Figure 2:**
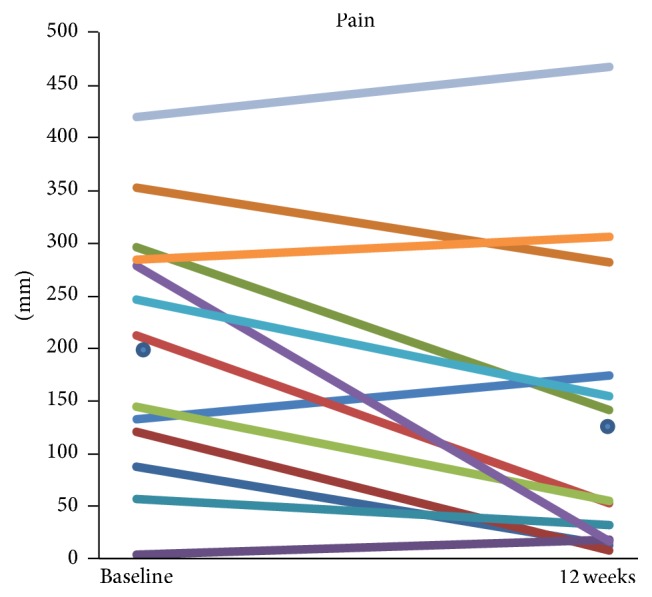
Individual changes in the WOMAC pain score in 12 weeks. The mean (SD) pain score at the baseline and 12-week time point is marked with a dot being 202 (123) mm and at 12-week time point 131 (143) mm, respectively (reduction 35%, *p* = 0.002).

Mean changes (95% CI) in the outcomes of interest are shown in Table 1 and Figure 1. Mean reduction in the WOMAC pain score was 35% (8% to 62%), with large individual variations; decline was seen in 9 of 13 participants (Figure 2). Reduction in the stiffness or function scores was also seen but did not reach statistical significance. The total WOMAC Index reduced by 27% (−4% to 57%, *p* = 0.079).

### 3.3. Effects on Physical Functioning

Mean SPPB score was 9.9 (1.2) at baseline, with no change at 12 weeks. Also, there were no significant changes in walking speed, chair stand, or step climbing times. Mean (95% CI) isometric leg extensor strength increased by 3.8 (1.1 to 6.6) N/body weight. Unexpectedly, mean TUG time was 1.4 s (0.6 to 2.2 s) slower at 12 weeks compared to baseline (Figure 1). Postural sway with eyes open showed a trend for small 6.3 (−0.3 to 12.9, *p* = 0.06) mm^2^/s increase in moment of velocity. Closing eyes, adding a cognitive task, and standing on foam increased sway and velocity compared with the eyes open test, with no statistically significant changes (results not shown). Hip extension ROM increased significantly, with the mean change being 30% (7% to 54%), but no significant changes were found in hip abduction or flexion. There was a trend for improvement in quality of life, with mean change of 13.8% (−2.4 to 29.9%, *p* = 0.09).

## 4. Discussion

The significant 30% reduction found in pain is large enough to be considered clinically relevant [9, 12]. Thus, the results of this pilot trial support and further develop the specific exercise program for rehabilitation of hip OA.

Besides pain, the purpose of the training was to improve joint function which was also largely achieved. Importantly, isometric leg extensor muscle strength improved statistically significantly by 20% and hip extension ROM by 30%. However, no improvements in ROM of the hip flexion or abduction were seen, possibly because both strengthening and ROM exercises were mainly targeted towards improving hip extension. Other outcomes of physical functioning remained unchanged or only showed a trend for improvement. Surprisingly, the TUG test even showed 15% worsening in spite of reduced pain and improved leg strength and hip ROM.

Hip OA may reduce postural stability, increasing the risk of falling. In our study, postural sway with eyes open increased slightly, as did hip extension ROM. This may indicate that the participants have better confidence in maintaining stability as a result of training, not necessarily declined balance [30]. Similarly, Nagy et al. showed greater sway in older adults after 8-week balance, strength, flexibility, and aerobic training in spite of improved functional performance. This might have been due to improved balance confidence related to trainees' better ability to control the motion of their hip and lower limbs [31]. It has also been shown that time of day effect in postural sway measurements is high especially in older adults [32]. In this study, the baseline and end point measurements were done at the same time of the day.

Wide individual variation in training responses and the small study sample possibly confounded some of the findings. OA is a disease with intermittent symptoms aggravated by various factors such as activity levels, lifestyle, and even time of day [33]. These may affect performance in mobility tests, such as the TUG and stair climbing. Therefore, in addition to a larger study group, a longer follow-up period with a control group with more than two measurement points is needed to evaluate the effects of exercise.

The recent Cochrane review by Fransen et al. demonstrated a significant improvement with exercise in self-reported pain and physical function among the small subset of participants with hip OA only, but the pain reduction was rather small [6]. Physical functioning was assessed objectively in only two studies [8, 9], and no between-group differences were observed. Results from more recent meta-analysis of land-based exercise for hip OA remain consistent with the Cochrane review. Similarly, water-based exercise therapy showed slight pain relief in patients with hip OA in the short term [34]. However, water-based exercise therapy is not always feasible. Also, no benefits of physical therapy either combined with exercise or alone have been found, both in terms of self-reported pain or function and in objectively measured changes in physical functioning [4, 34].

Whereas pain relief is the most important outcome, physical functioning ought to be evaluated objectively as well, because it is difficult to attain long-term benefits without clinically meaningful improvements in function. Twelve weeks is a short time for effective progression in training. Progressing too rapidly could worsen pain, likely discouraging patients to continue training. On the other hand, too light exercise may remain ineffective. In spite of the short duration, our results were encouraging. Most other exercise trials in hip OA patients have also been of short duration, mainly between 6 and 12 weeks, with some benefits reported immediately at the end of the intervention. Only two studies evaluated sustained benefits for physical function [7, 9], with neither demonstrating a significant reduction in pain. One reason for this may be the fact that the participants had relatively low WOMAC pain scores, with less room for improvement. This suggests that the frequency or intensity of exercise was too low or duration was too short to improve physical functioning.

Effective exercise in hip OA also requires motivated participants. Our training was started at low intensity and level of difficulty. Since group sizes were small, the leaders were able to pay individual attention to optimal joint loading and performance techniques to avoid aggravation of joint symptoms. This resulted in excellent training compliance over 12 weeks. Compliance may be more difficult to maintain over a longer duration.

## 5. Conclusions

Exercise programs focusing on improving aerobic capacity, quadriceps muscle strength, or lower extremity performance carried out 3 times weekly comprising at least 12 sessions have been considered optimal treatment for knee OA. These principles were followed in planning the exercise program for hip OA. The training program was found to be feasible and safe, though it was of a short duration. This study supports the use of exercise training in reducing hip OA pain. Further controlled studies with larger group sizes are needed to determine the long-term benefits of exercise and its effects on the progression of the disease.

## Figures and Tables

**Table 1:** Characteristics of the participants (mean (SD)).

	Baseline	End point
Daily walking, mean steps in 12 wks	5195 (2133)	NA
Mini-Mental State Examination Score (0–30)^1^	27.8 (2.3)	NA
Body fat, %^1^	42.5 (6.4)	NA
Femoral neck *t*-score^1, 2^	0.01 (0.93)	NA
WOMAC		
Total index (range: 0–2400)	796 (576)	583 (652)
Pain score (range: 0–500)	202.4 (123.4)	131.9 (143.6)
Stiffness score (range: 0–200)	99.1 (63.5)	76.8 (54.2)
Function score (range: 0–1700)	494.5 (413.9)	375.0 (474.1)
Physical functioning		
Normal walking speed, m/s	0.9 (0.2)	0.9 (0.2)
Fast walking speed, m/s	1.2 (0.2)	1.75 (1.8)
TUG, s	9.1 (1.5)	10.5 (2.2)
Chair stand time, s	14.8 (3.3)	14.2 (2.6)
Stair climb, s	11.5 (1.9)	12.2 (2.4)
Isometric leg extensor strength, N/kg	19.3 (8.0)	23.2 (10.2)
SPPB score (0–12)	9.9 (1.2)	9.9 (1.9)
Balance		
ML velocity, eyes open, mm/s	3.7 (2.3)	4.7 (2.5)
AP velocity, eyes open, mm/s	6.7 (2.8)	8.7 (6.3)
Moment of velocity, eyes open, mm^2^/s	8.8 (5.6)	15.1 (13.0)
ROM		
Hip abduction, arthritic side	33.2 (11.5)	33.8 (11.2)
Hip abduction, healthy side	42.0 (7.2)	42.6 (6.8)
Hip flexion, arthritic side	96.2 (10.2)	98.8 (14.2)
Hip flexion, healthy side	104.0 (10.7)	103.5 (11.4)
Hip extension, arthritic side	12.1 (4.5)	15.8 (5.9)
Hip extension, healthy side	16.8 (5.5)	19.5 (7.0)

^1^Only baseline measurements.

^2^Femoral neck bone density compared to reference population from Finland (age: 20–40 years).

**Table 2:** Detailed description of the training program.

Period	Description	Movements and Execution
Introduction to exercise hall, 2 weeks	Group training, weeks 1 and 2 (i) 10 min warm-up (ii) 20 min balance and agility exercises (iii) 20 min muscle strength, flexibility, and mobility exercises (iv) 10 min stretching	Warm-up and balance training in standing position Strength and mobility while training partly while sitting on a chair Stretching while sitting on a chair
I period: exercise hall, 5 weeks	Group training, weeks 3, 5, and 7 (i) 10 min warm-up (ii) 20 min balance and agility exercises (iii) 20 min muscle strength, flexibility, and mobility exercises (iv) 10 min stretching Circuit training, weeks 4 and 6: 8 movements, 1 min work, 1 min rest, 2 rounds (i) 10 min warm-up (ii) 40 min balance, agility, mobility, and muscle strengthening (iii) 10 min stretching	Balance and mobility: (i) Reducing base of support using different foot positions in standing (ii) Swaying, reaching out in different directions (iii) Changing directions and speed during walking, multidirectional stepping patterns (iv) Stepping over obstacles and using different surfaces for walking and stepping Muscle strength: (i) Knee extension in sitting position and flexion in standing position (ii) Hip abduction, flexion, and extension in standing position (iii) Sit to stand, squats, and heel raises (with or without support of a chair) (iv) Step board exercises with varying height (v) Trunk flexion and extension in sitting position (vi) Body weight, resistance bands, or ankle weights for resistance Flexibility and joint mobility: (i) Hip area, spine, upper limbs, and shoulder-neck region

Introduction to gym II period: in the gym, 4 weeks	Introduction, week 8 Circuit training in pairs, weeks 9–12 (i) 10–15 min warm-up emphasizing balance and mobility exercises (ii) 40 min training especially for the lower limbs (iii) 5–10 min stretching (iv) 6–8 exercises, 10–12 repetitions, 2 sets with 2 min rest (v) First 4 weeks, progression from the level 30% 1RM to 60% 1RM: target 60–65% 1RM (vi) Weekly 5–10% increase in resistance, accompanied by reduction in the number of repetitions	Flexibility and joint mobility: (i) Leg press (ii) Hip abduction (iii) Standing up from the chair (using a weight vest) (iv) Hip extension (v) Hip flexion (vi) Hip rotation (vii) Heel rise with a weight vest (viii) Back extension (ix) One limb chest press with body rotation (x) Rowing, sawing (xi) Squatting with pulley weights

## References

[1] Hochberg M. C., Altman R. D., April K. T. (2012). American College of Rheumatology 2012 recommendations for the use of nonpharmacologic and pharmacologic therapies in osteoarthritis of the hand, hip, and knee. *Arthritis Care & Research*.

[2] Zhang W., Nuki G., Moskowitz R. W. (2010). OARSI recommendations for the management of hip and knee osteoarthritis. Part III: changes in evidence following systematic cumulative update of research published through January 2009. *Osteoarthritis and Cartilage*.

[3] Arokoski J. P., Eskelinen A., Helminen E.-K. http://www.kaypahoito.fi/.

[4] Bennell K. L., Egerton T., Martin J. (2014). Effect of physical therapy on pain and function in patients with hip osteoarthritis: a randomized clinical trial. *JAMA*.

[5] Arokoski M. H., Arokoski J. P. A., Haara M. (2002). Hip muscle strength and muscle cross sectional area in men with and without hip osteoarthritis. *The Journal of Rheumatology*.

[6] Fransen M., McConnell S., Hernandez-Molina G., Reichenbach S. (2014). Exercise for osteoarthritis of the hip. *The Cochrane database of systematic reviews*.

[7] Fernandes L., Storheim K., Sandvik L., Nordsletten L., Risberg M. A. (2010). Efficacy of patient education and supervised exercise vs patient education alone in patients with hip osteoarthritis: a single blind randomized clinical trial. *Osteoarthritis and Cartilage*.

[8] French H. P., Cusack T., Brennan A. (2013). Exercise and manual physiotherapy arthritis research trial (EMPART) for osteoarthritis of the hip: a multicenter randomized controlled trial. *Archives of Physical Medicine and Rehabilitation*.

[9] Juhakoski R., Tenhonen S., Malmivaara A., Kiviniemi V., Anttonen T., Arokoski J. P. A. (2011). A pragmatic randomized controlled study of the effectiveness and cost consequences of exercise therapy in hip osteoarthritis. *Clinical Rehabilitation*.

[10] Tak E., Staats P., Van Hespen A., Hopman-Rock M. (2005). The effects of an exercise program for older adults with osteoarthritis of the hip. *The Journal of Rheumatology*.

[11] Carlson N. L., Christopherson Z., Arnall E. (2011). A pilot study of the effects of strength and aerobic conditioning in patients with hip osteoarthrotis. *Osteoarthritis and Cartilage*.

[12] Abbott J. H., Robertson M. C., Chapple C. (2013). Manual therapy, exercise therapy, or both, in addition to usual care, for osteoarthritis of the hip or knee: a randomized controlled trial. 1: clinical effectiveness. *Osteoarthritis and Cartilage*.

[13] Foley A., Halbert J., Hewitt T., Crotty M. (2003). Does hydrotherapy improve strength and physical function in patients with osteoarthritis—a randomised controlled trial comparing a gym based and a hydrotherapy based strengthening programme. *Annals of the Rheumatic Diseases*.

[14] Hopman-Rock M., Westhoff M. H. (2000). The effects of a health educational and exercise program for older adults with osteoarthritis of the hip or knee. *Journal of Rheumatology*.

[15] van Baar M. E., Dekker J., Oostendorp R. A. B. (1998). The effectiveness of exercise therapy in patients with osteoarthritis of the hip or knee: a randomized clinical trial. *The Journal of Rheumatology*.

[16] Fransen M., Nairn L., Winstanley J., Lam P., Edmonds J. (2007). Physical activity for osteoarthritis management: a randomized controlled clinical trial evaluating hydrotherapy or Tai Chi classes. *Arthritis Care & Research*.

[17] Juhl C., Christensen R., Roos E. M., Zhang W., Lund H. (2014). Impact of exercise type and dose on pain and disability in knee osteoarthritis: a systematic review and meta-regression analysis of randomized controlled trials. *Arthritis and Rheumatology*.

[18] Glyn-Jones S., Palmer A. J. R., Agricola R. (2015). Osteoarthritis. *The Lancet*.

[19] Bearne L. M., Walsh N. E., Jessep S., Hurley M. V. (2011). Feasibility of an exercise-based rehabilitation programme for chronic hip pain. *Musculoskeletal Care*.

[20] Uusi-Rasi K., Rauhio A., Kannus P. (2010). Three-month weight reduction does not compromise bone strength in obese premenopausal women. *Bone*.

[21] Bellamy N., Buchanan W. W., Goldsmith C. H., Campbell J., Stitt L. W. (1988). Validation study of WOMAC: a health status instrument for measuring clinically important patient relevant outcomes to antirheumatic drug therapy in patients with osteoarthritis of the hip or knee. *Journal of Rheumatology*.

[22] Soininen J. V., Paavolainen P. O., Gronblad M. A., Kaapa E. H. (2008). Validation study of a Finnish version of the Western Ontario and McMasters University osteoarthritis index. *HIP International*.

[23] Woolacott N. F., Corbett M. S., Rice S. J. C. (2012). The use and reporting of WOMAC in the assessment of the benefit of physical therapies for the pain of osteoarthritis of the knee: findings from a systematic review of clinical trials. *Rheumatology*.

[24] De Leo D., Diekstra R. F. W., Lonnqvist J. (1998). LEIPAD, an internationally applicable instrument to assess quality of life in the elderly. *Behavioral Medicine*.

[25] Podsiadlo D., Richardson S. (1991). The timed “Up and Go”: a test of basic functional mobility for frail elderly persons. *Journal of the American Geriatrics Society*.

[26] Guralnik J. M., Simonsick E. M., Ferrucci L. (1994). A short physical performance battery assessing lower extremity function: association with self-reported disability and prediction of mortality and nursing home admission. *Journals of Gerontology*.

[27] Rejeski W. J., Ettinger W. H., Schumaker S., James P., Burns R., Elam J. T. (1995). Assessing performance-related disability in patients with knee osteoarthritis. *Osteoarthritis and Cartilage*.

[28] Pua Y.-H., Wrigley T. W., Cowan S. M., Bennell K. L. (2008). Intrarater test-retest reliability of hip range of motion and hip muscle strength measurements in persons with hip osteoarthritis. *Archives of Physical Medicine and Rehabilitation*.

[29] Era P., Sainio P., Koskinen S., Haavisto P., Vaara M., Aromaa A. (2006). Postural balance in a random sample of 7,979 subjects aged 30 years and over. *Gerontology*.

[30] Pai Y.-C. (2003). Movement termination and stability in standing. *Exercise and Sport Sciences Reviews*.

[31] Nagy E., Feher-Kiss A., Barnai M., Domján-Preszner A., Angyan L., Horvath G. (2007). Postural control in elderly subjects participating in balance training. *European Journal of Applied Physiology*.

[32] Jorgensen M. G., Rathleff M. S., Laessoe U., Caserotti P., Nielsen O. B. F., Aagaard P. (2012). Time-of-day influences postural balance in older adults. *Gait & Posture*.

[33] Altman R., Alarcón G., Appelrouth D. (1991). The American college of rheumatology criteria for the classification and reporting of osteoarthritis of the hip. *Arthritis and Rheumatism*.

[34] Beumer L., Wong J., Warden S. J., Kemp J. L., Foster P., Crossley K. M. (2016). Effects of exercise and manual therapy on pain associated with hip osteoarthritis: a systematic review and meta-analysis. *British Journal of Sports Medicine*.

